# Hypoxia shifts activity of neuropeptide Y in Ewing sarcoma from growth-inhibitory to growth-promoting effects

**DOI:** 10.18632/oncotarget.1604

**Published:** 2013-11-26

**Authors:** Jason U. Tilan, Congyi Lu, Susana Galli, Ewa Izycka-Swieszewska, Joshua Patrick Earnest, Asim Shabbir, Lindsay M. Everhart, Shuo Wang, Samantha Martin, Meredith Horton, Akanksha Mahajan, David Christian, Alison O'Neill, Hongkun Wang, Tingting Zhuang, Magdalena Czarnecka, Michael D. Johnson, Jeffrey A. Toretsky, Joanna Kitlinska

**Affiliations:** ^1^ Department of Nursing, School of Nursing and Health Studies, Georgetown University, Washington DC; ^2^ Department of Human Science, School of Nursing and Health Studies, Georgetown University, Washington DC; ^3^ McGovern Institute, Massachusetts Institute of Technology, Boston, MA; ^4^ Department of Biochemistry and Molecular & Cellular Biology, Georgetown University Medical Center, Georgetown University, Washington DC; ^5^ Department of Pathology and Neuropathology, Medical University of Gdańsk, Poland; ^6^ Department of Oncology, Lombardi Comprehensive Cancer Center, Georgetown University Medical Center, Georgetown University, Washington DC; ^7^ Department of Biostatistics and Bioinformatics, Georgetown University Medical Center, Georgetown University, Washington DC

**Keywords:** Neuropeptide Y, Ewing sarcoma, hypoxia, cancer stem cells, angiogenesis

## Abstract

Ewing sarcoma (ES) is an aggressive malignancy driven by an oncogenic fusion protein, EWS-FLI1. Neuropeptide Y (NPY), and two of its receptors, Y1R and Y5R are up-regulated by EWS-FLI1 and abundantly expressed in ES cells. Paradoxically, NPY acting via Y1R and Y5R stimulates ES cell death. Here, we demonstrate that these growth-inhibitory actions of NPY are counteracted by hypoxia, which converts the peptide to a growth-promoting factor. In ES cells, hypoxia induces another NPY receptor, Y2R, and increases expression of dipeptidyl peptidase IV (DPPIV), an enzyme that cleaves NPY to a shorter form, NPY3-36. This truncated peptide no longer binds to Y1R and, therefore, does not stimulate ES cell death. Instead, NPY3-36 acts as a selective Y2R/Y5R agonist. The hypoxia-induced increase in DPPIV activity is most evident in a population of ES cells with high aldehyde dehydrogenase (ALDH) activity, rich in cancer stem cells (CSCs). Consequently, NPY, acting via Y2R/Y5Rs, preferentially stimulates proliferation and migration of hypoxic ALDHhigh cells. Hypoxia also enhances the angiogenic potential of ES by inducing Y2Rs in endothelial cells and increasing the release of its ligand, NPY3-36, from ES cells. In summary, hypoxia acts as a molecular switch shifting NPY activity away from Y1R/Y5R-mediated cell death and activating the Y2R/Y5R/DPPIV/NPY3-36 axis, which stimulates ES CSCs and promotes angiogenesis. Hypoxia-driven actions of the peptide such as these may contribute to ES progression. Due to the receptor-specific and multifaceted nature of NPY actions, these findings may inform novel therapeutic approaches to ES.

## INTRODUCTION

Ewing sarcoma (ES) is an aggressive malignancy affecting children and adolescents. The presence of metastases is the most adverse prognostic factor, with a 3-year event-free survival at 27% for patients with metastatic tumors [1]. This poor prognosis is associated with a frequent disease recurrence, suggesting that ES tumorigenic potential is driven by chemotherapy-resistant, metastatic cancer stem cells (CSCs). A population of ES cells with elevated aldehyde dehydrogenase (ALDH) activity is particularly rich in CSCs, as shown by its increased tumorigenic potential and chemoresistance [2].

The fraction of CSCs often increases in hypoxia, leading to disease dissemination and resistance to therapy [3-5]. In ES, the hypoxia-driven increase in tumor cell malignancy has been demonstrated using experimental models, while clinically tumor ischemia associates with unfavorable disease phenotype [6-11]. However, the mechanisms governing these effects remain unknown.

Malignant transformation of ES is driven by a chromosomal translocation resulting in fusion of the EWS gene with an ETS transcription factor, most often FLI1 [12]. Neuropeptide Y (NPY) and its Y1R and Y5R are among the transcriptional targets of EWS-FLI, highly expressed in ES tissues and cells [13-18]. However, microarray analysis revealed that mRNA of NPY's Y2R, which is not detectable in ES cells *in vitro*, is elevated in tissues from metastatic ES [18, 19]. Given the high release of NPY from many ES cell lines [17], this expression pattern raises a question about the role of the endogenous peptide in these tumors.

NPY is a 36 amino-acid neurotransmitter promoting proliferation and motility of various cells and regulating their differentiation [18, 20-26]. The peptide acts also as an angiogenic factor, stimulating endothelial cells (ECs) via their inducible Y2R [27]. Paradoxically, our data indicate that in ES, the EWS-FLI1-driven Y1R/Y5R/NPY autocrine loop stimulates tumor cell death [17, 18]. However, we have also shown that this effect of endogenous NPY is mitigated by dipeptidyl peptidase IV (DPPIV) expressed in ES cells [17]. This enzyme cleaves full length NPY_1-36_ to a shorter form, NPY_3-36_, which does not bind to Y1Rs and is unable to stimulate ES cell death [17, 28]. Importantly, NPY_3-36_ preserves the ability to activate Y2Rs and Y5Rs and becomes a selective Y2R/Y5R agonist [28]. Thus, given the known role of Y2R in angiogenesis and elevated levels of its mRNA in metastatic ES tissues [19, 29-33], we sought to determine the function of the Y2R pathway in these tumors.

Hypoxia, known to enhance ES malignancy, also regulates the NPY system. As elements of the angiogenic pathway, NPY, its Y2R and DPPIV, which converts the peptide to a Y2R/Y5R agonist, are up-regulated in ischemic tissues [31, 34-36]. Therefore, we hypothesized that Y2Rs, which are undetectable in ES cells *in vitro*, are induced in the tumor microenvironment by hypoxia. Indeed, we have shown that hypoxic conditions promote NPY-induced angiogenic processes and activate the Y2R/Y5R/DPPIV/NPY_3-36_ pathway in ES cells, changing the functions of the peptide from growth-inhibitory to growth-promoting activities. The latter is particularly apparent in ALDH^high^ CSCs, suggesting a potential role for NPY in promoting the malignant phenotype of hypoxic ES. These findings demonstrate for the first time the dynamic nature of NPY actions in ES and identify mechanisms by which tumor microenvironment converts the peptide to a growth-promoting factor, specific for tumorigenic ES CSCs. Our study also reveals a novel mechanism governing a hypoxia-induced increase in ES malignancy.

## RESULTS

### Y2R is expressed in endothelium and tumor cells from ES patient biopsies

**Fig 1:**
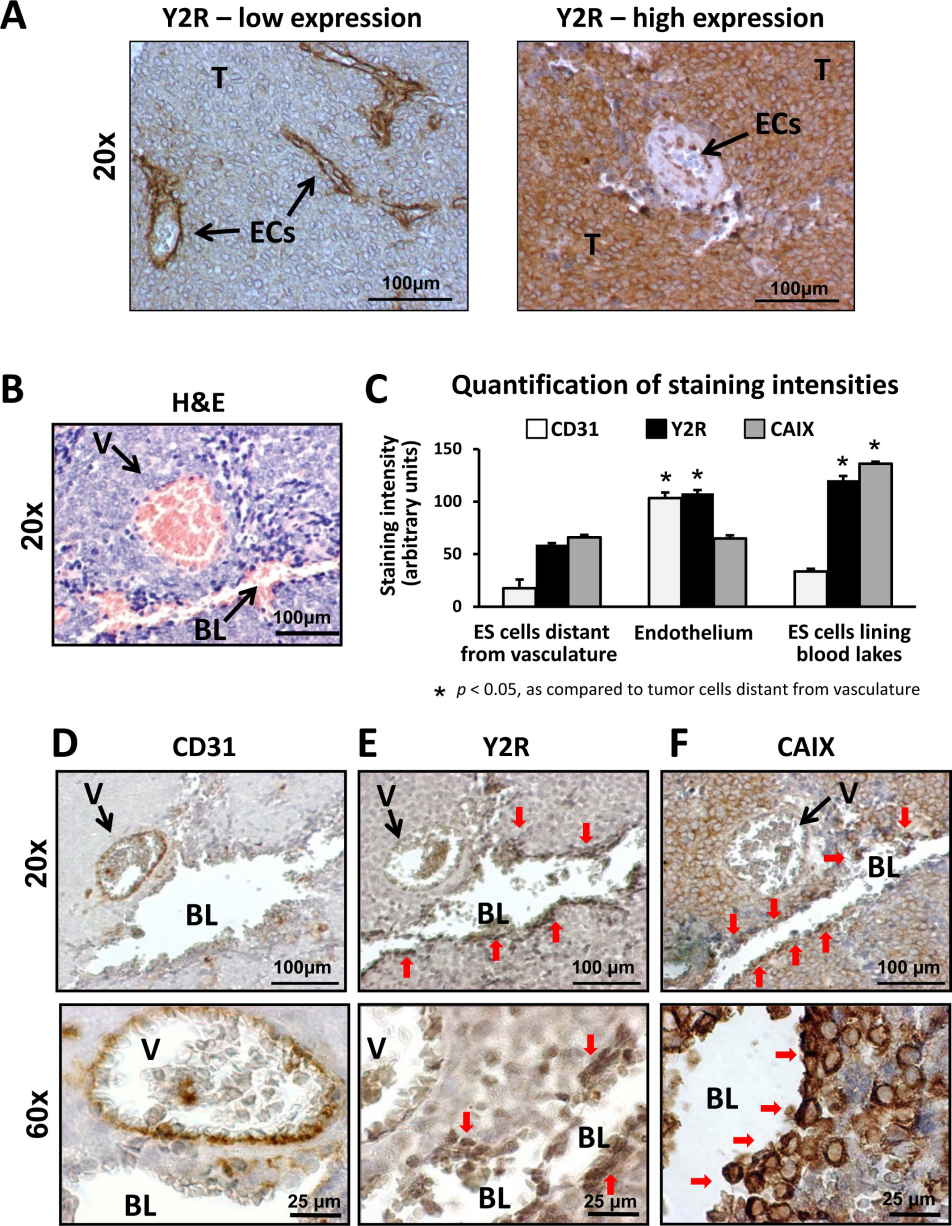
Human ES express Y2Rs in endothelium and tumor cells A. Y2R immunostaining in human ES tissue. ECs consistently express Y2R, while the percent of positive neoplastic cells varies between tumors. Based on the percent of Y2R-positive ES cells, tumors were categorized into low Y2R expression (0-10% of Y2R-positive ES cells) and high Y2R expression (40-100% of Y2R-positive cells). The representative images for both categories are shown. B, D-F. Serial sections of a tumor area with a typical blood vessel and blood lake stained with hematoxylin and eosin (H&E), anti-Y2R, anti-CD31 and anti-CAIX antibody. As shown by H&E staining (B), both structures are perfused, yet differentiated by positive and negative immunostaining for an endothelial marker, CD31, respectively (D). Tumor cells lining the blood lake lumen are positive for Y2R and a hypoxia marker, CAIX (E-F). C. The cellular populations within tumor tissues were divided into three categories: ES cells distant from vasculature (tumor cells within tumor parenchyma, not directly surrounding tumor vasculature or pseudo-vessels), endothelium (ECs within blood vessel lumen) and ES cells lining blood lakes (1-2 cellular layers demarcating blood lake lumen). Intensities of CD31, Y2R and CAIX immunoreactivity in particular cellular fractions were quantified and compared to the ES cells within tumor parenchyma. T- tumor cells; ECs – endothelial cells; BL – blood lake; V – blood vessel; red arrows indicate tumor cells lining lumen of blood lake

Y2R mRNA is elevated in metastatic ES tumors [19]. To identify the specific cells expressing this receptor, tissue sections from 16 human ES tumors were stained with anti-Y2R antibody (Supplementary Table 1). Y2R was present in tumor cells and ECs (Fig. 1A). While ECs uniformly expressed Y2R, the tumors varied in percent of Y2R-positive ES cells, dichotomizing into two groups – low Y2R (0-10% of tumor cells stained) and high Y2R (40-100% of Y2R-positive ES cells) (Fig. 1A). High Y2R expression tended to correlate with worse survival at 23 months post diagnosis (50% and 100% for patients with high and low Y2R, respectively) (Supplementary Fig. 1, Supplementary Table 1). Due to the small sample size, however, the difference did not reach statistical significance (p = 0.09).

In Y2R high tumors, intense Y2R staining was observed around blood lakes, which are perfused pseudovascular structures devoid of endothelial lining and therefore negative for endothelial marker, CD31 (Fig. 1B-E). In ES, such pseudo-vessels are formed by hypoxic tumor cells [10]. Consequently, staining of serial tumor sections revealed that the entire area surrounding blood lakes was positive for a hypoxia marker, CAIX (Fig. 1F) [37]. The cells with the highest CAIX expression were lining the lumens of blood lakes (Fig. 1C, F). The cells demarcating these pseudo-vessels were also highly positive for Y2R (Fig. 1C, E), suggesting preferential expression of this receptor in hypoxic ES cells.

### Hypoxia up-regulates the Y2R/Y5R/DPPIV/NPY_3-36_ system in ES cells

**Fig 2:**
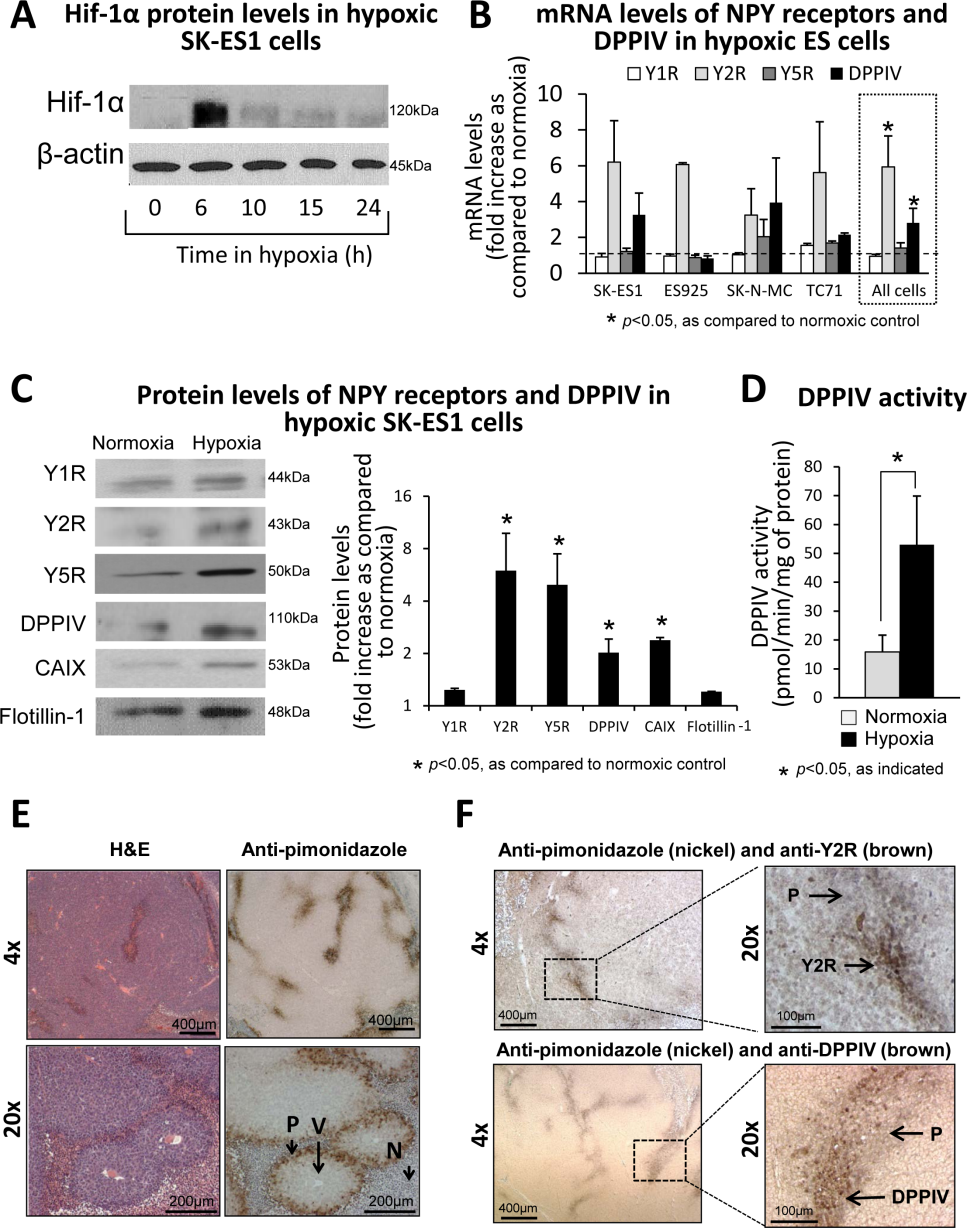
Hypoxia up-regulates the Y2R/Y5R/DPPIV system in ES cells A. Western blot analysis of Hif-1α expression in SK-ES1 cells cultured in 0.1% oxygen. B. mRNA levels of NPY receptors and DPPIV measured by real-time RT-PCR in four ES cell lines exposed to 0.1% oxygen for 12-24h. To identify genes universally elevated in hypoxic ES cells, the statistical analysis was performed on combined changes in all ES cell lines (dashed box). C. Western blot analysis of NPY receptor and DPPIV protein levels in cell membrane fractions from SK-ES1 cells cultured in normoxia or hypoxia for 24h. CAIX and flotillin-1 served as a hypoxia marker and loading control, respectively. Protein levels from three independent experiments were quantified by densitometry. D. Selective DPPIV activity was measured calorimetrically in SK-ES1 cells cultured for 24h in normoxia or hypoxia. E. SK-ES1 orthotopic xenografts were treated *in vivo* with an extrinsic hypoxia marker, pimonidazole, for 24h. Tumor sections were stained with H&E and anti-pimonidazole antibody. F. Double staining of the above SK-ES1 orthotopic xenografts with anti-pimonidazole (nickel) and anti-Y2R or anti-DPPIV (brown) antibodies. V – blood vessel; P- pimonidazole; N - necrosis.

To determine whether expression of Y2R is induced by hypoxia, ES cells were cultured in 0.1% oxygen, which stabilized Hif-1α within 6h (Fig. 2A). This hypoxic response was associated with changes in the NPY system. Y2R mRNA expression was induced in all ES cell lines tested, while DPPIV mRNA was increased in three out of four cell lines (Fig. 2B). When analyzed as combined changes in all tested ES cell lines, the increases in expression of Y2R and DPPIV were statistically significant, implicating these two genes as universal targets of hypoxia (Fig 2B). Levels of Y1R and Y5R mRNA remained unchanged. Consistently, in SK-ES1 cells, a model of NPY-rich ES [17], Western blot confirmed hypoxia-induced increases in Y2R and DPPIV protein levels, which was associated with elevated activity of the enzyme (Fig. 2C, D). Despite the lack of changes in Y5R mRNA, its protein levels were increased in hypoxic cells, while Y1R remained unchanged (Fig. 2C). Altogether, these hypoxia-induced changes suggested activation of the Y2R/Y5R/DPPIV axis.

### Y2R and DPPIV are expressed in hypoxic areas of ES tissue

To confirm the hypoxia-induced changes in the NPY system *in vivo*, we used an orthotopic ES xenograft model. Mice bearing SK-ES1 tumors were injected with a hypoxia probe, pimonidazole, and the tumors were harvested 24h later. Staining of tumor tissues with anti-pimonidazole antibody exhibited a characteristic pattern of hypoxic areas located near the necrotic regions, surrounding blood vessels at a distance of approximately 100 μm (Fig. 2E). Double-staining with anti-pimonidazole and anti-Y2R or anti-DPPIV antibody revealed accumulation of both proteins in hypoxic areas (Fig. 2F), supporting observations in human ES tissues (Fig. 1B-F).

### Hypoxia prevents the Y1R/Y5R-mediated cell death in ES cells and triggers a stimulatory effect of NPY on their anchorage-independent growth

**Fig 3:**
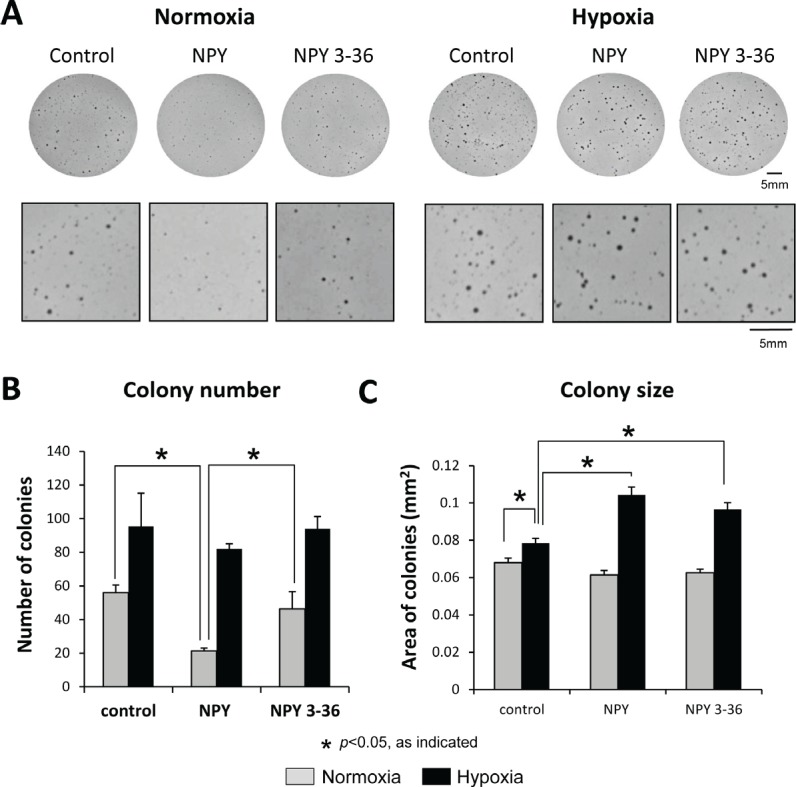
Hypoxia prevents NPY-driven cell death and triggers its stimulatory effect on anchorage-independent growth A. SK-ES1 cells were plated on soft agar in the presence or absence of full-length NPY or NPY_3-36_ (10^−7^M). Plates were incubated in normoxia or hypoxia for 14 days. Pictures of the representative wells for each treatment group are shown under low and high magnification. B. Average number of soft agar colonies per well in each experimental group. C. Average colony size under normoxic and hypoxic conditions

NPY stimulates ES cell death via the Y1R/Y5R pathway, while DPPIV counteracts this process by inactivating the peptide at Y1Rs [17, 18]. Since hypoxia up-regulated DPPIV, we sought to determine if this could prevent NPY-induced cell death. In normoxia, the full-length NPY, but not the product of DPPIV cleavage, NPY_3-36_, decreased the number of SK-ES1 soft agar colonies (Fig. 3A, B). This effect was not observed in hypoxia, the condition favoring DPPIV (Fig. 3A, B). Instead, hypoxia alone increased the size of SK-ES1 colonies, while both NPY and NPY_3-36_ administered under hypoxic conditions further augmented this effect (Fig. 3A, C). Since anchorage-independent growth depends on CSCs, and NPY_3-36_ selectively activates Y2R and Y5R, these results suggested a Y2R/Y5R-dependent increase in ES CSC proliferation.

### Y2R/Y5R/DPPIV system is preferentially activated in hypoxic ES CSCs

**Fig 4:**
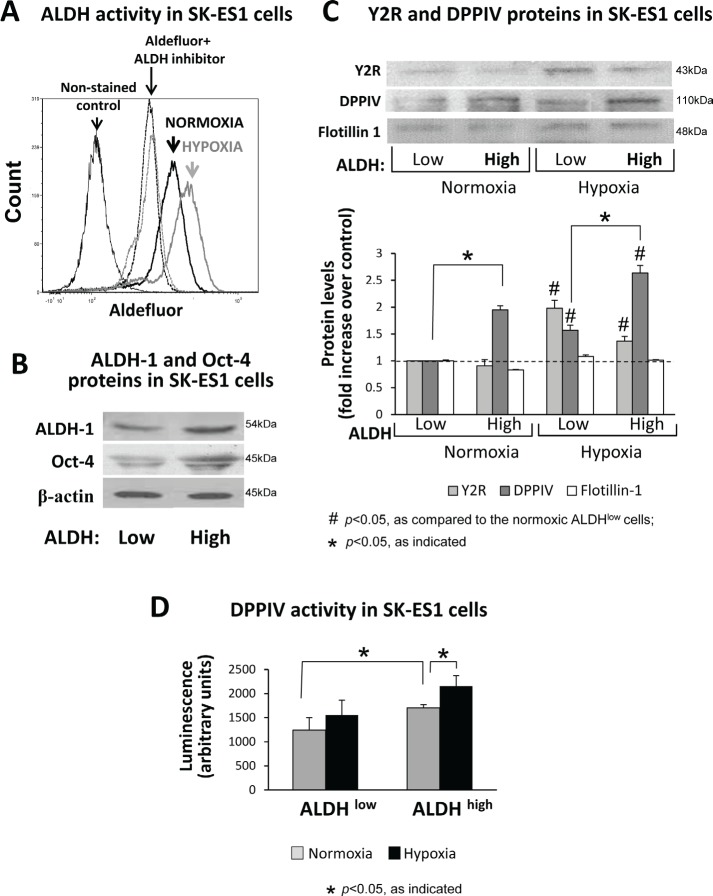
The Y2R/Y5R/DPPIV system is preferentially activated in ALDH^high^ ES CSCs A. SK-ES1 cells were cultured in normoxia or hypoxia for 24h, stained with Aldefluor, and ALDH activity was measured by FACS. The non-stained cells or the cells incubated with Aldefluor in the presence of ALDH inhibitor, DEAB, served as negative controls. B. SK-ES1 cells were stained with Aldefluor and FACS-sorted based on the ALDH activity into ALDH^high^ (upper 8% of cells) and ALDH^low^ (lower 10% of cells) cells. The levels of ALDH-1 protein and stem cell marker, Oct-4, were compared by Western blot in soluble protein fractions of ALDH^high^ and ALDH^low^ cells, to confirm the efficiency of cell sorting. C. ALDH^high^ and ALDH^low^ cells were cultured for 24h in normoxia and hypoxia. Protein levels of Y2Rs and DPPIV were detected in cell membrane fractions isolated from these cells by Western blot. Flotillin-1 served as a loading control. Protein levels from 4 independent experiments were quantified by densitometry. D. DPPIV activity was measured in the above cell membrane fractions via luminescent method.

The results of soft agar assay suggested that hypoxia-induced changes in the NPY system affect CSCs. Thus, in the subsequent experiments, we focused on ES cells with high ALDH activity, which have been shown to be rich in CSCs [2]. Culture under hypoxic conditions increased the number of SK-ES1 cells with elevated ALDH activity (Fig. 4A), suggesting hypoxia-driven enrichment in CSCs. To assess expression of the NPY system in ES CSC, SK-ES1 cells were sorted by flow cytometry into ALDH^high^ and ALDH^low^ cells (Supplementary Fig. 2). This approach allowed for testing two cell populations with maximal difference in ALDH activity. The efficiency of sorting was confirmed by elevated expression of one of the main forms of ALDH, ALDH1, and a stem cell marker, Oct-4, in ALDH^high^ cells (Fig. 4B). Western blot analysis of ALDH^low^ and ALDH^high^ cells cultured under normoxic and hypoxic conditions revealed hypoxia-induced up-regulation of Y2R protein in both cell fractions (Fig. 4C). However, in normoxia and hypoxia, expression of DPPIV was higher in ALDH^high^ cells, as compared to ALDH^low^ cells. Consequently, hypoxic CSCs had the highest DPPIV protein and activity levels (Fig. 4C, D).

### NPY stimulates proliferation and migration of hypoxic ES CSCs

Having established hypoxia-induced changes in NPY system expression, we sought to determine their functional consequences. To this end, SK-ES1 cells were sorted into a population of ALDH^high^ and ALDH^low^ cells as above. Although there were no significant differences in basal levels of the proliferation and migration between these cell fractions (data not shown), the cells varied in their responses to NPY.

**Fig 5:**
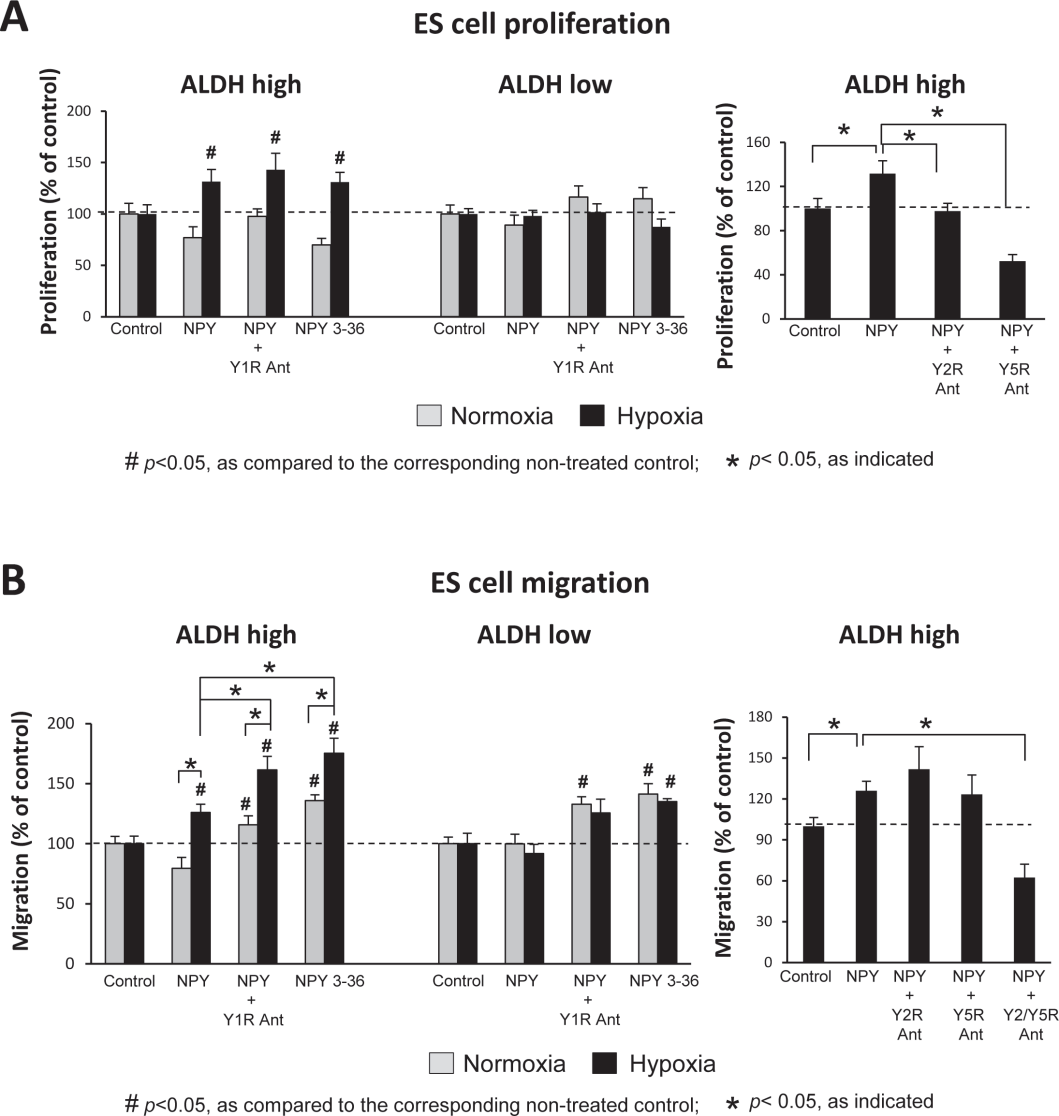
NPY stimulates proliferation and migration of hypoxic ALDH^high^ ES CSCs via the Y2R/Y5R pathway A. ALDH^high^ and ALDH^low^ cells were pre-incubated for 6h in normoxia or hypoxia and then treated under the same conditions with the desired NPY receptor agonists (10^−7^M) or antagonists (10^−6^M) for 12h. Cells were subsequently pulsed with 10μM EdU, and proliferation was measured as a percent of EdU positive cells. B. ALDH^high^ and ALDH^low^ cells were plated into BD FluoroBlok plates, treated as above for 18h and stained with Calcein AM. Cell migration was assessed based on fluorescence measured from the bottom of the plate.

In normoxia, NPY had no significant effect on the proliferation of ALDH^high^ and ALDH^low^ SK-ES1 cells (Fig. 5A). However, in hypoxia, the peptide selectively stimulated proliferation of ALDH^high^ cells. This effect of NPY in hypoxic ALDH^high^ cells was mimicked by Y2R/Y5R-specific treatment - the full-length NPY in the presence of Y1R antagonist or a Y2R/Y5R selective agonist, NPY_3-36_ (Fig. 5A). Consequently, both Y2R and Y5R antagonists reduced the mitogenic effect of NPY in hypoxic ALDH^high^ cells (Fig. 5A).

Similar to its proliferative actions, full-length NPY stimulated migration preferentially in hypoxic ADLH^high^ SK-ES1 cells (Fig. 5B). Treatment with NPY in the presence of Y1R antagonist or with NPY_3-36_ further enhanced the NPY-induced SK-ES1 cell migration in hypoxia. This Y2R/Y5R-specific treatment also moderately increased migration of normoxic ALDH^high^ and ALDH^low^ cells (Fig. 5B). The effect of NPY in hypoxic ALDH^high^ cells was blocked by the combination of Y2R and Y5R antagonist (Fig. 5B). Altogether, these results demonstrate the Y2R/Y5R-mediated mitogenic and pro-migratory activity of NPY, which is triggered by hypoxia favoring the Y2R/Y5R/DPPIV system in ES CSCs.

### Hypoxia enhances the ability of ES cells to stimulate angiogenesis via release of NPY

**Fig 6:**
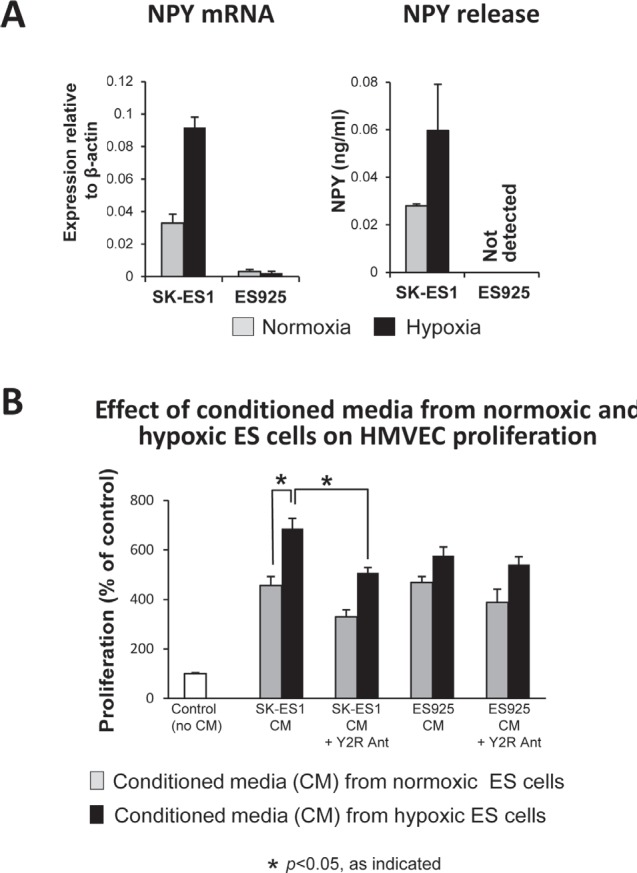
Hypoxia augments the ability of ES cells to trigger angiogenesis via release of NPY A. SK-ES1 and ES925 cells were cultured in normoxia or hypoxia for 24h. NPY mRNA in ES cells was measured by real-time RT-PCR, while ELISA was used to measure NPY concentrations in their conditioned media. B. HMVEC cells were growth-arrested and treated with conditioned media from hypoxic and normoxic ES cells. HMVEC proliferation was measured by [^3^H]thymidine uptake

NPY is an angiogenic factor acting via its Y2R, the expression of which is induced in ECs by hypoxia or exposure to NPY [27, 31]. The peptide has been previously implicated in the vascularization of ES tumors [18]. To determine if its angiogenic effect is enhanced by hypoxia, we used two ES cell lines varying in NPY release [17]. In SK-ES1 cells, which constitutively express high levels of NPY, 24h culture in hypoxia further increased NPY synthesis and release (Fig. 6A). No increase in NPY expression was observed in ES925 cells, which do not secrete the peptide under basal conditions (Fig. 6A).

To determine functional consequences of these changes, we tested the effect of conditioned media from normoxic and hypoxic ES cells on HMVEC proliferation. The proliferative effects of conditioned media from normoxic SK-ES1 and ES925 were comparable and not dependent on NPY, since Y2R antagonist did not significantly decrease them (Fig. 6B). Conditioned media from hypoxic SK-ES1 cells exerted a higher proliferative effect, as compared to the media from normoxic cells (Fig. 6B). This hypoxia-induced increase in HMVEC proliferation was blocked by Y2R, proving its dependency on NPY. No statistically significant increase in proliferative activity of conditioned media from hypoxic ES925 cells was observed, consistent with the lack of NPY release from these cells (Fig. 6A, B).

### Hypoxia sensitizes ECs to NPY released from ES cells

**Fig 7:**
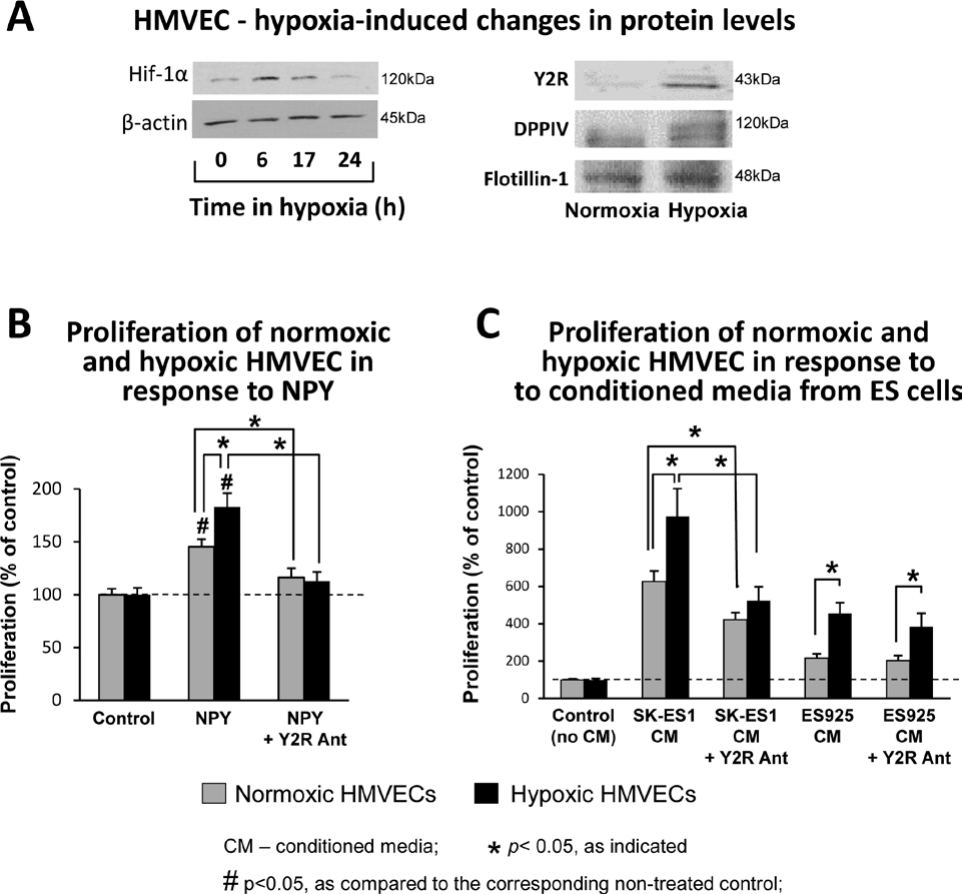
Hypoxia sensitizes ECs to NPY A. HMVECs were cultured in 0.1% oxygen. Hif-1α protein was detected in soluble protein fractions by Western blot at desired time points, while Y2R and DPPIV proteins were measured in cell membrane preparations upon 24h incubation in normoxia or hypoxia. B. HMVECs were growth arrested for 24h under normoxia or hypoxia and treated for the following 24h with NPY (10^−7^M) with or without Y2R antagonist (10^−6^M) at the respective oxygen concentrations. Proliferation was measured by [^3^H]thymidine uptake. C. HMVECs were pretreated as above, stimulated with conditioned media from ES cells cultured under basal conditions, and incubated in normoxia or hypoxia for the following 24h. Proliferation was measured as above.

Having established the hypoxia-induced increase in NPY release from ES cells, we sought to determine if its angiogenic activity can be augmented by parallel changes in ECs. HMVECs were cultured in 0.1% oxygen, which up-regulated Hif-1α within 6h (Fig. 7A). After 24h, hypoxia induced Y2R, which was not detectable in normoxic HMVECs (Fig. 7A). Simultaneously, hypoxia increased DPPIV protein levels (Fig. 7A) and augmented its activity up to 140% of the normoxic control (data not shown). This hypoxia-induced activation of the Y2R/DPPIV system sensitized HMVECs to NPY. The mitogenic activity of the peptide was elevated in hypoxic HMVECs, as compared to the normoxic cells (Fig. 7B). In normoxia and hypoxia, NPY effects were blocked by Y2R antagonist. Similarly, NPY-rich SK-ES1 conditioned media exerted enhanced proliferative activity in hypoxic HMVECs, as compared to normoxic cells (Fig. 7C). Y2R antagonist blocked this effect, thereby confirming its dependency on activation of the Y2R/DPPIV system in ECs. The proliferative activity of ES925 conditioned media, which lacks NPY, was also higher in hypoxic HMVECs (Fig. 7C). However, Y2R antagonist did not block this increase.

### Y2R antagonist reduces vascularization of ES xenografts

**Fig 8:**
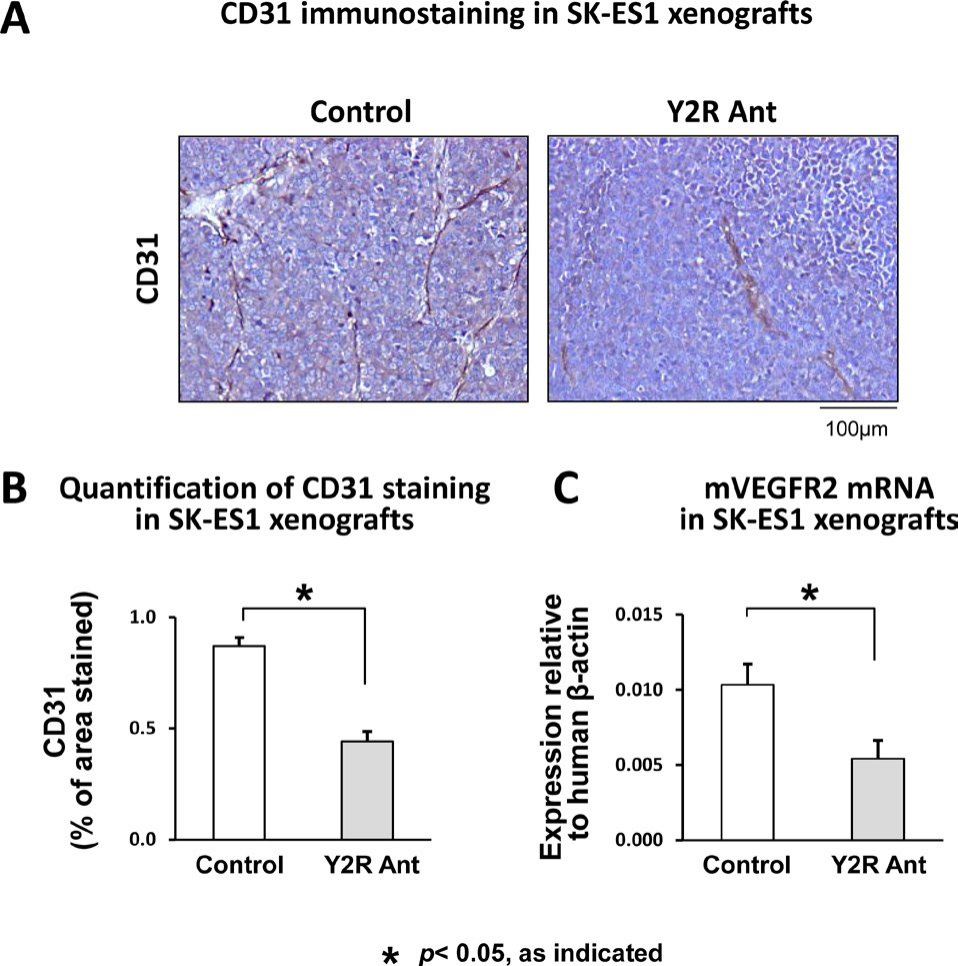
Y2R antagonist impairs vascularization of ES xenografts A. Representative images of immunostaining for endothelial marker, CD31, in SK-ES1 subcutaneous xenografts treated with placebo or Y2R antagonist (10^−6^M). B. Tumor vascularization of control and Y2R antagonist-treated SK-ES1 xenografts measured by area of CD31 staining. C. mRNA levels of mouse VEGFR2 measured in SK-ES1 xenografts by real-time RT-PCR

Our observations *in vitro* suggested that the angiogenic activity of NPY in ES is induced in tumor microenvironment by hypoxia. To confirm this, SK-ES1 subcutaneous xenografts were treated with Y2R antagonist. This blockage of Y2R resulted in significant decrease in tumor vascularization, as measured by area of CD31-positive endothelial cells (Fig. 8A, B). This effect was associated with reduced mRNA levels of mouse vascular endothelial growth factor receptor 2 (VEGFR2) (Fig. 8C). Altogether, these results confirm the role of NPY and its Y2R in ES vascularization.

## DISCUSSION

Growing evidence indicates a role of hypoxia in ES progression. In ES patients, tumor ischemia is associated with an unfavorable metastatic pattern, while its absence correlates with better prognosis [11]. *In vitro*, hypoxia up-regulates EWS-FLI1 protein and modifies its transcriptional profile, promoting genes involved in tumor progression [6]. Hypoxic ES cells exhibit an enhanced anchorage-independent growth, invasiveness, and resistance to apoptosis induced by exogenous factors [6-9, 38]. However, the mechanisms of these effects remain unclear. In fact, some data indicate no hypoxia-induced changes in ES cell proliferation, but rather an increase in apoptosis when the cells are assayed as a whole population in adherent culture [6, 7]. Thus, such a hypoxia-induced increase in tumor malignancy may be driven by specific changes in CSCs.

CSCs are resistant to low oxygen and their fraction increases in hypoxia-treated tumor cells [3-5]. In our study, hypoxic conditions increased the population of ES cells with high ALDH activity, which are rich in tumorigenic CSCs. We propose that this enrichment in ES CSCs in combination with activation of the Y2R/Y5R/DPPIV/NPY_3-36_ system in these cells is one of the driving mechanisms governing the hypoxia-driven increase in ES malignancy.

NPY, and its Y1R and Y5R are transcriptional targets of EWS-FLI1 up-regulated in ES [13, 14]. Paradoxically, we have previously shown that this Y1R/Y5R/NPY autocrine loop stimulates ES cell death [17, 18]. However, mRNA of another NPY receptor, Y2R, is up-regulated in metastatic ES tissues [19]. Since Y2R is not detectable in cultured ES cells [18], the goal of our study was to determine its localization and function in ES. Y2R was present in ECs, but also in tumor cells, and observed to accumulate in hypoxic areas. Consistently, we have shown that Y2R expression is induced in ES cells by hypoxia. This effect was associated with increases in NPY release, Y5R protein levels, and in expression of DPPIV, an enzyme converting the peptide to a selective Y2R/Y5R agonist. These changes suggested an overall hypoxia-induced activation of the Y2R/Y5R/DPPIV/NPY_3-36_ axis. Similar up-regulation of the NPY system was reported in ischemic tissues, while DPPIV was elevated in various hypoxic tumor cells [31, 34-36].

ES cells are rich in Y1R, which is expressed in response to EWS-FLI1 [13-16, 18]. However, in hypoxia, Y1R expression did not change, while levels of Y2R and Y5R increased, altering the NPY receptor ratio. Moreover, increased DPPIV activity, such as that observed in hypoxic ES cells, alters the form of released NPY to NPY_3-36_, a selective Y2R/Y5R agonist [17]. Altogether, these changes prevent binding of NPY to Y1Rs and block ES cell death mediated by the Y1R/Y5R/NPY_1-36_ pathway. Furthermore, DPPIV-induced cleavage of NPY enables the activation of moderately expressed Y2Rs and Y5Rs in the presence of abundant Y1Rs. We have shown that while Y2Rs are induced in both ALDH^high^ and ALDH^low^, DPPIV is preferentially up-regulated in ALDH^high^ cells. Our results are in agreement with reports of DPPIV serving as a marker of metastatic CSCs in other tumors [39].

Consistent with preferential DPPIV activation in CSCs, NPY stimulated the proliferation of hypoxic ALDH^high^ cells, but not ALDH^low^ cells. The fact that either Y2R or Y5R alone blocked this effect suggested that activation of both receptors is required to trigger it. The selective mitogenic activity of NPY in ALDH^high^ cells is consistent with reports indicating differential effects of hypoxia and NPY on ES cells, depending on culture conditions. While both factors increase the size of CSC-dependent soft agar colonies, they have no effect on the proliferation of non-sorted, adherent cells, comprised mostly of non-CSCs [6, 7, 18].

As observed for its proliferative effect, full-length NPY stimulated migration only in hypoxic ALDH^high^ cells. However, treatment with NPY in the presence of Y1R antagonist or with NPY_3-36_ alone augmented this effect and moderately increased cell migration in normoxic ALDH^high^ and ALDH^low^ cells. These observations suggest that in normoxia, selective activation of Y5Rs is sufficient to stimulate ES cell migration, while hypoxia-induced expression of Y2Rs further augments this process. Consequently, both Y2R and Y5R antagonists were required to block the pro-migratory effects of NPY in hypoxic ALDH^high^ cells, suggesting that independent activation of one of these receptors promotes cell migration. Altogether, our data indicate that hypoxia-induced changes in NPY functions are particularly apparent in a CSC-rich ALDH^high^ cells. These changes may contribute to the increase in ES metastatic properties, albeit without an effect on the primary tumor growth that is dependent mostly on non-CSCs. Future studies with animal models will explore this hypothesis.

The angiogenic activity of NPY depends on Y2R [30]. In hypoxic NPY-rich ES cells, simultaneous increases in NPY and DPPIV expression led to elevated release of the peptide, presumably as a Y2R/Y5R-selective agonist, NPY_3-36_. This, in turn, increased the angiogenic activity of ES conditioned media. This stimulatory effect was further enhanced by increases in Y2R and DPPIV expression in hypoxic ECs, which sensitized them to NPY's proliferative effect. Consequently, Y2R antagonist significantly reduced vascularization of NPY-rich ES xenografts. Y2R expression in ECs within human ES tissues supported the clinical relevance of these findings.

The elevated responsiveness of hypoxic ECs to ES conditioned media lacking NPY implicated a role for other ES-derived angiogenic factors, such as VEGF [40]. Importantly, the NPY and VEGF angiogenic systems are interdependent, with NPY serving as an upstream factor [31, 41]. Consistently, Y2R antagonist decreased expression of VEGFR2 in NPY-rich SK-ES1 xenografts, suggesting that VEGF pathway activation is NPY-dependent. Importantly, SK-ES1 cells have low basal VEGF levels and lack mechanisms that mediate its hypoxia-induced up-regulation in other ES cells [40]. Therefore, in the subset of such VEGF-insufficient tumors, hypoxia-induced activation of the NPY system is essential to maintain tumor vascularization by direct stimulation of ECs and up-regulation of VEGF system.

NPY receptor patterns in ES and ECs are similar, with constitutively expressed Y1R and inducible Y2R [17, 27]. Consequently, in both cases, DPPIV-induced NPY cleavage is essential to enable activation of Y2Rs, which are expressed at lower levels than abundant Y1Rs. These observations suggest a potential for multifunctional therapies for ES. Since the same Y2R/Y5R/DPPIV/NPY_3-36_ system is responsible for hypoxia-induced proliferative and pro-migratory effects of NPY in ECs and ES CSCs, blocking this pathway *in vivo* may target both metastatic and angiogenic properties of the tumors. This has been shown in neuroblastoma, where Y2R antagonist reduced xenograft growth via inhibition of tumor cell proliferation and vascularization [27].

In addition to the typical vasculature, ES tumors are known to form pseudo-vessels, also called blood lakes, which augment blood flow to the tumor tissue. An increased presence of these structures is associated with poor prognosis in ES patients [42]. The formation of pseudo-vessels is driven by hypoxia [10]. Consistently, the tumor cells surrounding them are positive for hypoxia markers, as well as Y2R. These observations warrant further investigation as to the potential role for Y2R and NPY in vascular mimicry.

**Fig 9:**
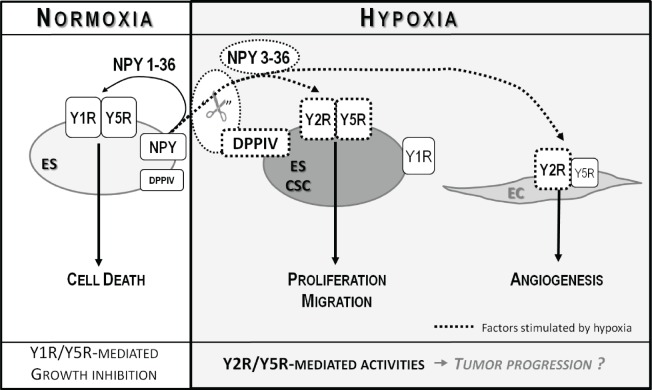
Model of NPY actions in ES In normoxia, NPY is released as a full-length peptide and stimulates Y1/Y5R-mediated ES cell death. Hypoxia increases expression of Y2R, Y5R and DPPIV in ES CSCs. These changes lead to cleavage of NPY and selective activation of Y2R/Y5Rs, which promotes proliferation and migration of these tumorigenic cells. NPY_3-36_, a product of DPPIV cleavage, stimulates also Y2Rs expressed in hypoxic ECs and promotes angiogenesis. Altogether, these hypoxia-induced actions may promote tumor progression

Altogether, our results revealed the dynamic nature of NPY actions in ES and critical role of the tumor microenvironment in their regulation. The net effect of endogenous NPY on ES depends on the balance between Y1R/Y5R/NPY_1-36_ growth-inhibitory effects on the entire population of ES cells and Y2R/Y5R/DPPIV/NPY_3-36_ proliferative and pro-migratory effects on ES CSCs and ECs (Fig. 9). Hypoxia shifts this balance toward growth-promoting processes by preventing activation of Y1Rs and promoting the Y2R/Y5R pathway. Further studies are required to determine the impact of these changes in the NPY system on ES progression and metastases.

Despite the complexity of NPY functions in ES, the receptor specificity of its actions and potential for multifaceted therapeutic effects make the peptide and its receptors attractive targets in ES treatment. We have shown that exogenous NPY inhibits ES xenograft growth via Y1R/Y5R-mediated cell death [17, 18]. Thus, receptor specific interventions activating the Y1R/Y5R pathway, inhibiting Y2R/Y5Rs, and/or targeting DPPIV may provide new therapeutic opportunities for ES patients. Recently, a variety of selective NPY receptor agonists and antagonists have been developed. This includes Y1R/Y5R selective agonists and various Y2R antagonists [43]. Some of them, such as DPP inhibitors and Y5R antagonist are already being used in clinical practice or trials for other disorders [44-46]. Importantly, however, our previous studies indicate that prolonged treatment of ES xenografts with DPP inhibitors results in increased DPPIV expression, which overcomes their effects [17]. While this could possibly be circumvented by changes in drug regimen or its potency, targeting NPY receptors may be a better therapeutic strategy. This approach is also more specific, given the high range of known DPPIV substrates.

In summary, we have shown for the first time fundamental changes that can occur in the NPY/DPPIV system within the tumor microenvironment and their functional consequences. Our findings highlight the importance of assessing NPY functions in tissue environment, uncover its novel role in cancer stem cells and reveal mechanisms governing a hypoxia-induced increase in tumor malignancy. Although our study focuses on ES, hypoxia-induced activation of the Y2R/Y5R/ DPPIV/NPY_3-36_ axis may be applicable to other tumors rich in NPY and its receptors. A similar pattern of NPY receptor expression and its effect on tumor cell proliferation and migration has been reported in breast cancer [21, 23]. The evidence of a role for both NPY and DPPIV in a variety of other tumors is also growing [18, 27, 47-49]. Thus, understanding the NPY system may direct new therapeutic opportunities for patients with metastatic ES, thus far lacking an effective treatment, as well as those with other malignancies.

## METHODS

### Materials

NPY and NPY_3-36_ were purchased from Bachem (San Carlos, CA); Y1R antagonist, BIBP 3226, from Sigma (St. Louis, MO); Y5R antagonist, CGP71683, and Y2R antagonist, BIIE0246, from Tocris (Ellisville, MO). DPPIV inhibitor, Sitagliptin, was a gift from Dr. Bachovchin (Tufts University, Boston, MA).

Human tissue sections from 16 ES archival paraffin embedded samples were collected from multiple institutions in Poland by one of the co-authors (EIS) in compliance with institutional ethical regulations. Use of these samples was approved by Georgetown University Institutional Review Board.

### ES xenografts

For the orthotopic xenografts, SK-ES1 cells were injected into gastrocnemius muscles of SCID/bg mice [50]. Once tumors reached 1cm^3^, mice were injected intraperitoneally with a hypoxia probe, pimonidazole (HPI, Burlington, MA), 1.5mg/mouse, and tumors were harvested 24h later. For the subcutaneous xenografts, SK-ES1 cells were injected into nude mice and treated daily with local injections of Y2R antagonist (10^−6^M) [17, 27]. Treatment was initiated when the tumors reached a volume of approximately 100mm^3^, and the experiment was terminated 2 weeks later. Tumor vascularization was quantified by immunohistochemistry, as area of positive staining for endothelial marker, CD31, while expression of mouse vascular endothelial growth factor receptor 2 (VEGFR2) was measured by real-time RT-PCR.

### Immunohistochemistry

Immunohistochemistry was performed using the following antibodies: rabbit polyclonal anti-Y2R (Neuromics, Edina, MN), anti-CD26 (Novus Biologicals, LLC, Littleton, CO), anti-pimonidazole (Hydroxyprobe^TM^-1 kit) (HPI, Inc. Burlington, MA), anti-carbonic anhydrase IX (CAIX) and anti-mouse CD31 (Abcam, Cambridge, MA) and mouse monoclonal anti-human CD31 (DAKO, Carpinteria, CA). Staining intensity was quantified using Metamorph software.

### Cell culture

Human ES cells were obtained and cultured as previously reported [17]. Human dermal microvascular endothelial cells (HMVEC) purchased from Lonza (Basel, Switzerland) were cultured according to the supplier's recommendation. Hypoxia was created in a chamber containing 0.1% O_2_, 95% N_2_ and 5% CO_2_.

Real time RT-PCR and NPY ELISA were performed as previously described [17, 18].

### Aldefluor assay and cell sorting

ES cells were stained using ALDEFLUOR kit (Stemcell Technologies, Vancouver, Canada). Fluorescence-activated cell sorting (FACS) was performed on FACSAria (BD, Franklin Lakes, NJ) utilizing FACSDiva and FCS Express 4 softwares (DeNovo Software, Los Angeles, CA). Cells were sorted into ALDH^high^ (upper 8%) and ALDH^low^ cells (lower 10%), cultured for no longer than 24h in normoxia or hypoxia, and assayed as desired. ALDH activity in normoxia and hypoxia was measured by FACS upon 24h culture.

### Cell membrane isolation and Western blot

Cell lysates were fractionated into membrane and soluble proteins, as previously described [51]. Western blot was performed using the following antibodies: rabbit polyclonal anti-Y1R (gift from Dr. Urban, University of Chicago), anti-DPPIV and anti-CAIX (Abcam, Cambridge, MA), anti-flotilllin-1 (Sigma, St. Louis, MO) and anti-hypoxia-inducible factor 1α (Hif-1α) (Cell Signaling Technologies, Danvers, MA), goat polyclonal anti-Npy2r (My Biosource, Camarillo, CA) and anti-Y5R (Everest Biotech, Ramona, CA) and mouse monoclonal anti-ALDH1 (BD Biosciences, San Jose, CA), anti-Oct-4 (Santa Cruz Biotechnology, Santa Cruz, CA) and anti-β-actin (Sigma, St. Louis, MO).

DPP activity from unsorted cells was measured calorimetrically, as previously described [17], while for sorted ES cells DPPIV-Glo™ Protease Assay (Promega, Madison, WI) was used. DPPIV activity was calculated as a fraction of DPP activity blocked by its selective inhibitor, Sitagliptin (10^−5^M) [17].

Colony formation on soft agar was performed as previously described [17]. Cells were treated with NPY or NPY_3-36_ (10^−7^M) and incubated in normoxia or hypoxia for 14 days.

ES cell proliferation was measured using Click-iT® EdU cell proliferation assay (Life Technologies, Grand Island, NY). ALDH^high^ and ALDH^low^ ES cells were pre-incubated in normoxia or hypoxia for 6h and treated with NPY (10^−7^M) +/− receptor antagonists (10^−6^M) or with NPY_3-36_ (10^−7^M) for 12h. Cells were pulsed with EdU, fixed and fluorescently labeled according to the manufacturer's procedure. Proliferation was measured as a percent of EdU-positive cells.

### ES cell migration

ALDH^high^ and ALDH^low^ cells were plated onto BD FluoroBlok™ 96-well plates (BD Biosciences, San Jose, CA), pre-incubated for 6h in hypoxia or normoxia and treated as above for 18h. Cells were stained with Calcein AM and fluorescence was measured from the bottom of the plate.

### HMVEC proliferation

ES cells were grown in HMVEC culture media, which was collected 24h later. HMVECs were plated onto 96-well plates, growth-arrested for 24h, and then treated for 24h with NPY (10^−8^M) or ES-conditioned media, +/− Y2R antagonist (10^−6^M). Proliferation was measured using [^3^H] thymidine [18].

### Statistical analysis

Statistical analysis was performed using SigmaStat® and GraphPad softwares. One-way repeated measure ANOVA with post-hoc t-test (P<0.05) using Dunnett's method was used for data comparison and analysis. All experiments were repeated at least three times. Data is presented as mean ± standard errors.

## Supplementary Tables and Figures





## References

[R1] Ladenstein R, Potschger U, Le Deley MC, Whelan J, Paulussen M, Oberlin O, van den Berg H, Dirksen U, Hjorth L, Michon J, Lewis I, Craft A, Jurgens H (2010). Primary disseminated multifocal Ewing sarcoma: results of the Euro-EWING 99 trial. J Clin Oncol.

[R2] Awad O, Yustein JT, Shah P, Gul N, Katuri V, O'Neill A, Kong Y, Brown ML, Toretsky JA, Loeb DM (2010). High ALDH activity identifies chemotherapy-resistant Ewing's sarcoma stem cells that retain sensitivity to EWS-FLI1 inhibition. PLoS One.

[R3] Toffoli S, Michiels C (2008). Intermittent hypoxia is a key regulator of cancer cell and endothelial cell interplay in tumours. The FEBS journal.

[R4] Zhou J, Zhang Y (2008). Cancer stem cells: Models, mechanisms and implications for improved treatment. Cell cycle (Georgetown, Tex.

[R5] Das B, Tsuchida R, Malkin D, Koren G, Baruchel S, Yeger H (2008). Hypoxia enhances tumor stemness by increasing the invasive and tumorigenic side population fraction. Stem cells (Dayton, Ohio).

[R6] Aryee DN, Niedan S, Kauer M, Schwentner R, Bennani-Baiti IM, Ban J, Muehlbacher K, Kreppel M, Walker RL, Meltzer P, Poremba C, Kofler R, Kovar H (2010). Hypoxia modulates EWS-FLI1 transcriptional signature and enhances the malignant properties of Ewing's sarcoma cells in vitro. Cancer research.

[R7] Knowles HJ, Schaefer KL, Dirksen U, Athanasou NA (2010). Hypoxia and hypoglycaemia in Ewing's sarcoma and osteosarcoma: regulation and phenotypic effects of Hypoxia-Inducible Factor. BMC cancer.

[R8] Batra S, Reynolds CP, Maurer BJ (2004). Fenretinide cytotoxicity for Ewing's sarcoma and primitive neuroectodermal tumor cell lines is decreased by hypoxia and synergistically enhanced by ceramide modulators. Cancer research.

[R9] Kilic M, Kasperczyk H, Fulda S, Debatin KM (2007). Role of hypoxia inducible factor-1 alpha in modulation of apoptosis resistance. Oncogene.

[R10] van der Schaft DW, Hillen F, Pauwels P, Kirschmann DA, Castermans K, Egbrink MG, Tran MG, Sciot R, Hauben E, Hogendoorn PC, Delattre O, Maxwell PH, Hendrix MJ, Griffioen AW (2005). Tumor cell plasticity in Ewing sarcoma, an alternative circulatory system stimulated by hypoxia. Cancer research.

[R11] Dunst J, Ahrens S, Paulussen M, Burdach S, Jurgens H (2001). Prognostic impact of tumor perfusion in MR-imaging studies in Ewing tumors. Strahlenther Onkol.

[R12] Toomey EC, Schiffman JD, Lessnick SL (2010). Recent advances in the molecular pathogenesis of Ewing's sarcoma. Oncogene.

[R13] Smith R, Owen LA, Trem DJ, Wong JS, Whangbo JS, Golub TR, Lessnick SL (2006). Expression profiling of EWS/FLI identifies NKX2.2 as a critical target gene in Ewing's sarcoma. Cancer Cell.

[R14] Hancock JD, Lessnick SL (2008). A transcriptional profiling meta-analysis reveals a core EWS-FLI gene expression signature. Cell cycle (Georgetown, Tex.

[R15] Korner M, Waser B, Reubi JC (2008). High expression of neuropeptide Y1 receptors in ewing sarcoma tumors. Clin Cancer Res.

[R16] van Valen F, Winkelmann W, Jurgens H (1992). Expression of functional Y1 receptors for neuropeptide Y in human Ewing's sarcoma cell lines. J Cancer Res Clin Oncol.

[R17] Lu C, Tilan JU, Everhart L, Czarnecka M, Soldin SJ, Mendu DR, Jeha D, Hanafy J, Lee CK, Sun J, Izycka-Swieszewska E, Toretsky JA, Kitlinska J (2011). Dipeptidyl Peptidases as Survival Factors in Ewing Sarcoma Family of Tumors: IMPLICATIONS FOR TUMOR BIOLOGY AND THERAPY. The Journal of biological chemistry.

[R18] Kitlinska J, Abe K, Kuo L, Pons J, Yu M, Li L, Tilan J, Everhart L, Lee EW, Zukowska Z, Toretsky JA (2005). Differential effects of neuropeptide Y on the growth and vascularization of neural crest-derived tumors. Cancer research.

[R19] Schaefer KL, Eisenacher M, Braun Y, Brachwitz K, Wai DH, Dirksen U, Lanvers-Kaminsky C, Juergens H, Herrero D, Stegmaier S, Koscielniak E, Eggert A, Nathrath M, Gosheger G, Schneider DT, Bury C (2008). Microarray analysis of Ewing's sarcoma family of tumours reveals characteristic gene expression signatures associated with metastasis and resistance to chemotherapy. Eur J Cancer.

[R20] Hansel DE, Eipper BA, Ronnett GV (2001). Neuropeptide Y functions as a neuroproliferative factor. Nature.

[R21] Medeiros PJ, Al-Khazraji BK, Novielli NM, Postovit LM, Chambers AF, Jackson DN (2011). Neuropeptide Y stimulates proliferation and migration in the 4T1 breast cancer cell line. International journal of cancer.

[R22] Pons J, Kitlinska J, Jacques D, Perreault C, Nader M, Everhart L, Zhang Y, Zukowska Z (2008). Interactions of multiple signaling pathways in neuropeptide Y-mediated bimodal vascular smooth muscle cell growth. Can J Physiol Pharmacol.

[R23] Sheriff S, Ali M, Yahya A, Haider KH, Balasubramaniam A, Amlal H (2010). Neuropeptide Y Y5 receptor promotes cell growth through extracellular signal-regulated kinase signaling and cyclic AMP inhibition in a human breast cancer cell line. Mol Cancer Res.

[R24] Han R, Kitlinska JB, Munday WR, Gallicano GI, Zukowska Z (2012). Stress hormone epinephrine enhances adipogenesis in murine embryonic stem cells by up-regulating the neuropeptide Y system. PLoS One.

[R25] Son MY, Kim MJ, Yu K, Koo DB, Cho YS (2011). Involvement of neuropeptide Y and its Y1 and Y5 receptors in maintaining self-renewal and proliferation of human embryonic stem cells. Journal of cellular and molecular medicine.

[R26] Lee NJ, Doyle KL, Sainsbury A, Enriquez RF, Hort YJ, Riepler SJ, Baldock PA, Herzog H (2010). Critical role for Y1 receptors in mesenchymal progenitor cell differentiation and osteoblast activity. J Bone Miner Res.

[R27] Lu C, Everhart L, Tilan J, Kuo L, Sun CC, Munivenkatappa RB, Jonsson-Rylander AC, Sun J, Kuan-Celarier A, Li L, Abe K, Zukowska Z, Toretsky JA, Kitlinska J (2010). Neuropeptide Y and its Y2 receptor: potential targets in neuroblastoma therapy. Oncogene.

[R28] Mentlein R (1999). Dipeptidyl-peptidase IV (CD26)--role in the inactivation of regulatory peptides. Regul Pept.

[R29] Ekstrand AJ, Cao R, Bjorndahl M, Nystrom S, Jonsson-Rylander AC, Hassani H, Hallberg B, Nordlander M, Cao Y (2003). Deletion of neuropeptide Y (NPY) 2 receptor in mice results in blockage of NPY-induced angiogenesis and delayed wound healing. Proc Natl Acad Sci U S A.

[R30] Lee EW, Grant DS, Movafagh S, Zukowska Z (2003). Impaired angiogenesis in neuropeptide Y (NPY)-Y2 receptor knockout mice. Peptides.

[R31] Lee EW, Michalkiewicz M, Kitlinska J, Kalezic I, Switalska H, Yoo P, Sangkharat A, Ji H, Li L, Michalkiewicz T, Ljubisavljevic M, Johansson H, Grant DS, Zukowska Z (2003). Neuropeptide Y induces ischemic angiogenesis and restores function of ischemic skeletal muscles. J Clin Invest.

[R32] Tilan JU, Everhart LM, Abe K, Kuo-Bonde L, Chalothorn D, Kitlinska J, Burnett MS, Epstein SE, Faber JE, Zukowska Z (2013). Platelet neuropeptide Y is critical for ischemic revascularization in mice. FASEB J.

[R33] Koulu M, Movafagh S, Tuohimaa J, Jaakkola U, Kallio J, Pesonen U, Geng Y, Karvonen MK, Vainio-Jylha E, Pollonen M, Kaipio-Salmi K, Seppala H, Lee EW, Higgins RD, Zukowska Z (2004). Neuropeptide Y and Y2-receptor are involved in development of diabetic retinopathy and retinal neovascularization. Ann Med.

[R34] Dang DT, Chun SY, Burkitt K, Abe M, Chen S, Havre P, Mabjeesh NJ, Heath EI, Vogelzang NJ, Cruz-Correa M, Blayney DW, Ensminger WD, St Croix B, Dang NH, Dang LH (2008). Hypoxia-inducible factor-1 target genes as indicators of tumor vessel response to vascular endothelial growth factor inhibition. Cancer research.

[R35] Raghuraman G, Kalari A, Dhingra R, Prabhakar NR, Kumar GK (2010). Enhanced neuropeptide Y synthesis during intermittent hypoxia in the rat adrenal medulla: role of reactive oxygen species-dependent alterations in precursor peptide processing. Antioxid Redox Signal.

[R36] Eltzschig HK, Faigle M, Knapp S, Karhausen J, Ibla J, Rosenberger P, Odegard KC, Laussen PC, Thompson LF, Colgan SP (2006). Endothelial catabolism of extracellular adenosine during hypoxia: the role of surface adenosine deaminase and CD26. Blood.

[R37] Ebbesen P, Pettersen EO, Gorr TA, Jobst G, Williams K, Kieninger J, Wenger RH, Pastorekova S, Dubois L, Lambin P, Wouters BG, Van Den Beucken T, Supuran CT, Poellinger L, Ratcliffe P, Kanopka A (2009). Taking advantage of tumor cell adaptations to hypoxia for developing new tumor markers and treatment strategies. Journal of enzyme inhibition and medicinal chemistry.

[R38] Magwere T, Burchill SA (2011). Heterogeneous role of the glutathione antioxidant system in modulating the response of ESFT to fenretinide in normoxia and hypoxia. PLoS One.

[R39] Pang R, Law WL, Chu AC, Poon JT, Lam CS, Chow AK, Ng L, Cheung LW, Lan XR, Lan HY, Tan VP, Yau TC, Poon RT, Wong BC (2010). A subpopulation of CD26+ cancer stem cells with metastatic capacity in human colorectal cancer. Cell stem cell.

[R40] McCarty G, Awad O, Loeb DM (2011). WT1 protein directly regulates expression of vascular endothelial growth factor and is a mediator of tumor response to hypoxia. The Journal of biological chemistry.

[R41] Medeiros PJ, Jackson DN (2013). Neuropeptide Y Y5-receptor activation on breast cancer cells acts as a paracrine system that stimulates VEGF expression and secretion to promote angiogenesis. Peptides.

[R42] Sun B, Zhang S, Zhao X, Zhang W, Hao X (2004). Vasculogenic mimicry is associated with poor survival in patients with mesothelial sarcomas and alveolar rhabdomyosarcomas. Int J Oncol.

[R43] Walther C, Morl K, Beck-Sickinger AG (2011). Neuropeptide Y receptors: ligand binding and trafficking suggest novel approaches in drug development. J Pept Sci.

[R44] Erondu N, Gantz I, Musser B, Suryawanshi S, Mallick M, Addy C, Cote J, Bray G, Fujioka K, Bays H, Hollander P, Sanabria-Bohorquez SM, Eng W, Langstrom B, Hargreaves RJ, Burns HD (2006). Neuropeptide Y5 receptor antagonism does not induce clinically meaningful weight loss in overweight and obese adults. Cell metabolism.

[R45] Erondu N, Wadden T, Gantz I, Musser B, Nguyen AM, Bays H, Bray G, O'Neil PM, Basdevant A, Kaufman KD, Heymsfield SB, Amatruda JM (2007). Effect of NPY5R antagonist MK-0557 on weight regain after very-low-calorie diet-induced weight loss. Obesity (Silver Spring, Md.

[R46] Lambeir AM, Scharpe S, De Meester I (2008). DPP4 inhibitors for diabetes--what next?. Biochem Pharmacol.

[R47] Gilaberte Y, Roca MJ, Garcia-Prats MD, Coscojuela C, Arbues MD, Vera-Alvarez JJ (2012). Neuropeptide Y expression in cutaneous melanoma. Journal of the American Academy of Dermatology.

[R48] Massoner P, Kugler KG, Unterberger K, Kuner R, Mueller LA, Falth M, Schafer G, Seifarth C, Ecker S, Verdorfer I, Graber A, Sultmann H, Klocker H (2013). Characterization of Transcriptional Changes in ERG Rearrangement-Positive Prostate Cancer Identifies the Regulation of Metabolic Sensors Such as Neuropeptide Y. PLoS One.

[R49] Thompson MA, Ohnuma K, Abe M, Morimoto C, Dang NH (2007). CD26/dipeptidyl peptidase IV as a novel therapeutic target for cancer and immune disorders. Mini reviews in medicinal chemistry.

[R50] Merchant MS, Yang X, Melchionda F, Romero M, Klein R, Thiele CJ, Tsokos M, Kontny HU, Mackall CL (2004). Interferon gamma enhances the effectiveness of tumor necrosis factor-related apoptosis-inducing ligand receptor agonists in a xenograft model of Ewing's sarcoma. Cancer research.

[R51] Pfeiffer M, Koch T, Schroder H, Klutzny M, Kirscht S, Kreienkamp HJ, Hollt V, Schulz S (2001). Homo- and heterodimerization of somatostatin receptor subtypes. Inactivation of sst(3) receptor function by heterodimerization with sst(2A). The Journal of biological chemistry.

