# Supplementary material for: Hypoxia shifts activity of neuropeptide Y in Ewing sarcoma from growth-inhibitory to growth-promoting effects
Source: Oncotarget. 2013 Nov 26;4(12):2487–501. doi: 10.18632/oncotarget.1604 (PMC3926843; doi:10.18632/oncotarget.1604)
Supplement: Supplementary file 1 [file oncotarget-04-2487-s001.pdf]

Hypoxia shifts activity of neuropeptide Y in Ewing sarcoma from growth-inhibitory to growth-promoting effects – Tilan et al

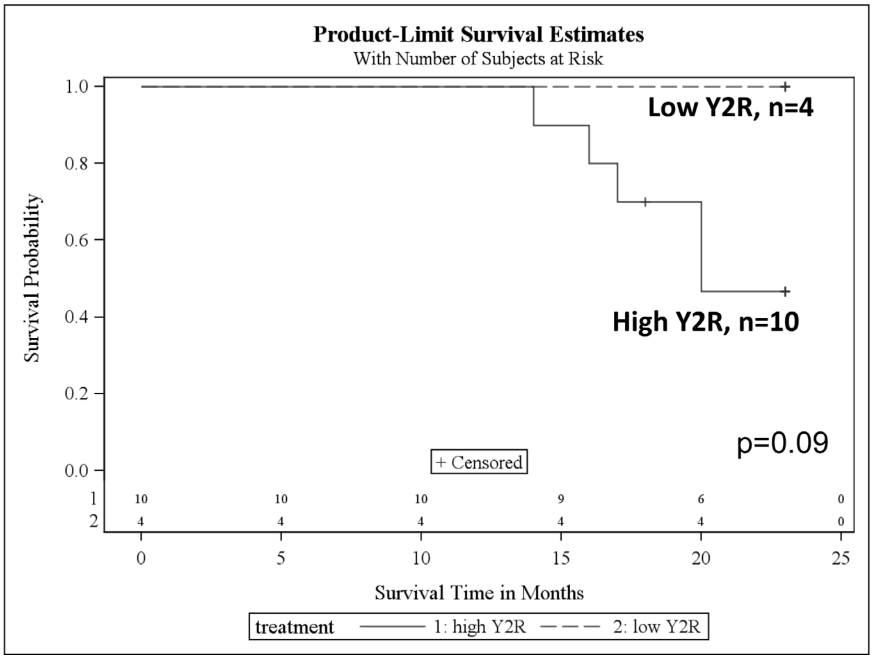

Supplementary figure 1: High Y2R expression in tumor cells tends to correlate with worse prognosis in ES patients. Human ES tumors were categorized based on the percent of Y2R-positive tumor cells into low Y2R expression (0-10% of Y2R-positive ES cells) and high Y2R expression (40-100% of Y2R-positive cells). The graph represents survival of ES patients as a function of Y2R expression.

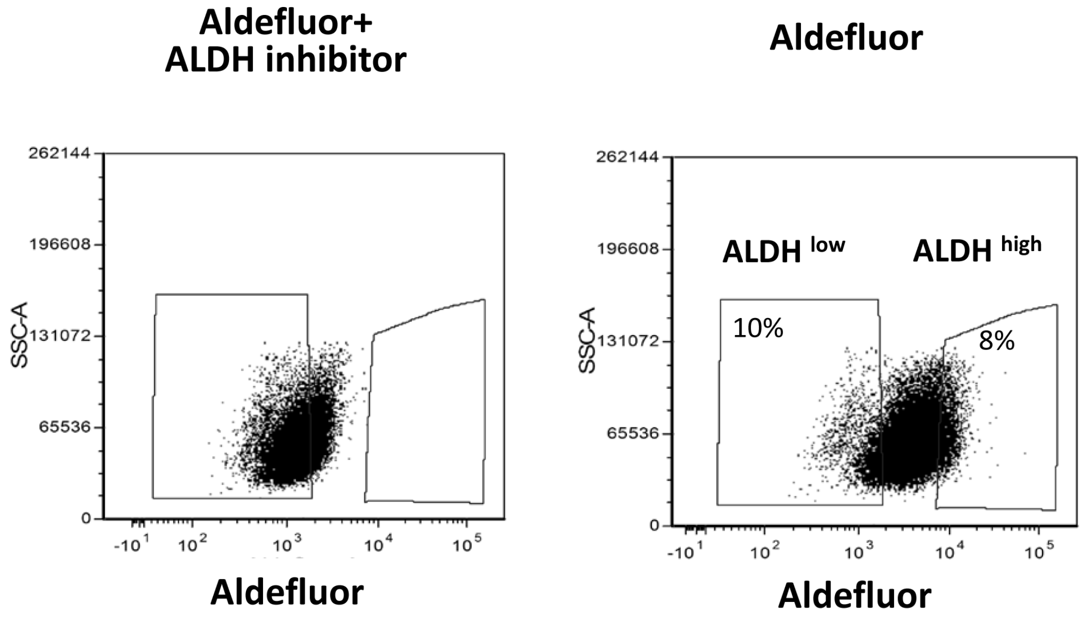

Supplementary figure 2: FACS sorting of Aldefluor-labeled SK-ES1 cells. SK-ES1 cells were stained with Aldefluor, and ALDH activity measured by FACS. Non-stained cells or cells incubated with Aldefluor in the presence of ALDH inhibitor, DEAB, served as negative controls. Based on ALDH activity, cells were FACS-sorted into two populations of ALDHhigh (upper 8% of cells) and ALDHlow (lower 10% of cells) cells.
