# Supplementary material for: Hypoxia shifts activity of neuropeptide Y in Ewing sarcoma from growth-inhibitory to growth-promoting effects
Source: Oncotarget. 2013 Nov 26;4(12):2487–501. doi: 10.18632/oncotarget.1604 (PMC3926843; doi:10.18632/oncotarget.1604)
Supplement: Supplementary file 2 [file oncotarget-04-2487-s002.pdf]

Hypoxia shifts activity of neuropeptide Y in Ewing sarcoma from growth-inhibitory to growth-promoting effects – Tilan et al

Supplementary table 1: Comparison of clinical features and tumor characteristics in ES patients with low and high percent of Y2R-positive tumor cells.

	**Y2R low**<br>(0-10% of Y2R+ tumor cells)<br>n=6	**Y2R high**<br>(40-100% of Y2R+ tumor cells)<br>n=10
**Gender**		
Male	2 (33%)	5 (50%)
Female	4 (67%)	5 (50%)
**Age at diagnosis (years)**		
Median	10.5	13
Range	7-31	2-38
**Tumor localization**		
Bone	5 (83%)	6 (60%)
Soft tissue	1 (17%)	4 (40%)
**Stage at diagnosis**		
Localized	5 (83%)	8 (80%)
Metastatic	1 (17%)	2 (20%)
**Blood lakes**		
Present	5 (83%)	10 (100%)
Not present	1 (17%)	0 (0%)
**Average % of Y2R positive cells around blood lakes**	13%	70%
**Percent survival at 23 months**	100%*	50% **p=0.09**

* Survival data available for 4 out of 6 patients
